# Long-Term IoT-Based Maternal Monitoring: System Design and Evaluation

**DOI:** 10.3390/s21072281

**Published:** 2021-03-24

**Authors:** Fatemeh Sarhaddi, Iman Azimi, Sina Labbaf, Hannakaisa Niela-Vilén, Nikil Dutt, Anna Axelin, Pasi Liljeberg, Amir M. Rahmani

**Affiliations:** 1 Department of Computing, University of Turku, 20014 Turku, Finland; iman.azimi@utu.fi (I.A.); pasi.liljeberg@utu.fi (P.L.); 2 Department of Computer Science, University of California, Irvine, CA 92697-3435, USA; slabbaf@uci.edu (S.L.); dutt@uci.edu (N.D.); a.rahmani@uci.edu (A.M.R.); 3 Department of Nursing Science, University of Turku, 20520 Turku, Finland; hmniel@utu.fi (H.N.-V.); anmaax@utu.fi (A.A.); 4 School of Nursing, University of California, Irvine, CA 92697, USA; 5 Institute for Future Health (IFH), University of California, Irvine, CA 92697, USA

**Keywords:** Internet of Things, wearable device, maternal health, remote health monitoring

## Abstract

Pregnancy is a unique time when many mothers gain awareness of their lifestyle and its impacts on the fetus. High-quality care during pregnancy is needed to identify possible complications early and ensure the mother’s and her unborn baby’s health and well-being. Different studies have thus far proposed maternal health monitoring systems. However, they are designed for a specific health problem or are limited to questionnaires and short-term data collection methods. Moreover, the requirements and challenges have not been evaluated in long-term studies. Maternal health necessitates a comprehensive framework enabling continuous monitoring of pregnant women. In this paper, we present an Internet-of-Things (IoT)-based system to provide ubiquitous maternal health monitoring during pregnancy and postpartum. The system consists of various data collectors to track the mother’s condition, including stress, sleep, and physical activity. We carried out the full system implementation and conducted a real human subject study on pregnant women in Southwestern Finland. We then evaluated the system’s feasibility, energy efficiency, and data reliability. Our results show that the implemented system is feasible in terms of system usage during nine months. We also indicate the smartwatch, used in our study, has acceptable energy efficiency in long-term monitoring and is able to collect reliable photoplethysmography data. Finally, we discuss the integration of the presented system with the current healthcare system.

## 1. Introduction

Maternity care aims to ensure the health and well-being of both the pregnant woman and her fetus. Maternal health has a great impact on the infant during the pregnancy but also in the future. In addition, health complications during pregnancy, for example, hypertensive disorders or gestational diabetes, may resonate with corresponding health problems in the pregnant woman’s later life [1,2,3]. Hence, maternity care is essential to promote long-term health at the population level as well as preventing acute pregnancy complications in individuals. Regular check-up during pregnancy is essential to detect abnormalities and to prevent additional complications, injuries or even death [4].

Traditionally, blood pressure, blood glucose and urine tests have been the main concrete parameters to follow during pregnancy, as well as the growth of the uterus and maternal weight gain. To support a healthy lifestyle, maternity care providers also need to provide counseling about other lifestyle and self-management matters, for example, physical activity and sleep. However, these are not yet systematically monitored [5]. There is a need to ubiquitously monitor pregnant women’s health to early detect possible complications and improve health parameters [6,7,8]. Moreover, continuous monitoring of different health parameters enables gaining fine-grained quantitative data that could provide a better understanding of pregnancy.

Technological advancements in Information and Communication Technology (ICT) are transforming the way healthcare is delivered. In particular, the IoT is an emerging paradigm in modern ICT that exploits various sensing, communication, and computing infrastructures to offer an advanced network of objects anywhere and anytime [9,10,11]. This paradigm, coupled with big data analytics and artificial intelligence, can be incorporated into healthcare services, providing remote health monitoring for individuals 24/7. Such a monitoring system can collect data from the user and her/his environment, transmit the data to remote servers, analyze the data and provide recommendations and feedback accordingly.

IoT-based systems can provide cost-efficient health monitoring services for pregnant women in everyday settings [12]. Recent studies show that such remote health monitoring systems can improve health outcomes for both mother and baby during pregnancy and the postpartum [13,14].

Many attempts have thus far been conducted to provide remote health monitoring for pregnant women. Several studies leverage subjective methods, where mothers are inquired about their health and well-being [15,16,17]. These methods are mostly restricted to scheduled phone-interviews and Internet-based questionnaires, which might be inaccurate [18]. In other studies, various parameters such as blood pressure and weight are periodically collected from pregnant women at home. These works are also bounded to limited data collection [19,20]. In addition, mobile applications and wearable electronics are utilized to continuously collect health parameters during and after pregnancy, targeting specific pregnancy-related issues such as sleep disturbances, physical activity and hypertension [21,22,23].

Even though the existing works in the literature employ IoT-based systems to perform remote maternal monitoring, they are narrowly focused on a specific health problem, equipped with limited sensing capabilities and, most notably, tested in a short period of time during pregnancy. Moreover, implementation challenges of such maternal long-term IoT-based systems have not investigated thus far. Feasibility of a mobile application during pregnancy [20] and a wristband during pregnancy and postpartum [6] have been studied in the literature.

To be able to operate for a long period of time, data collection in an IoT-based monitoring system needs to address several key implementation challenges including: (1) feasibility and usability; (2) energy consumption and efficiency; and (3) reliability and accuracy. Deficiency in delivering any of these characteristics results in diminished users’ quality of of experience [6,24,25,26,27,28,29].

In this paper, we present a long-term IoT-based health monitoring system to continuously and remotely offer various services during pregnancy and postpartum. Our system employs heterogeneous subjective and objective data collection techniques to track mothers’ health status. Subsequently, the data are stored and analyzed remotely, and the processed data are delivered to the health providers. We carried out a full system implementation for a real human subject study providing health monitoring during pregnancy and postpartum. The implemented system allows the monitoring of stress, sleep and physical activity of pregnant women. We also integrated various AI-based and machine learning methods into the system in a holistic way, providing a data analysis pipeline. This pipeline contains deep learning-based quality assessment of data [30], personalized modeling, missing data imputation and anomaly detection [21,31]. We then evaluate and discuss the challenges in implementing and deploying the presented remote maternal monitoring system. In summary, the contributions of this paper are as follows:

- We present a feasible long-term IoT-based maternal monitoring system used during pregnancy and postpartum. We investigate both system level and user level requirements (e.g., energy efficiency and feasibility) to enhance user experience.
- We implemented a proof-of-concept monitoring system for a real human subject study.
- We analyzed and evaluated the challenges in implementing such monitoring systems, including feasibility, reliability, energy efficiency and integration of the presented system with the current healthcare system.
- We integrated AI-based methods previously proposed by the authors into the presented system in a holistic way for analyzing the data and providing monitoring services.

The rest of this paper is organized as follows. In Section 2, we outline the state-of-the-art long-term maternal monitoring. In Section 3, we describe monitoring services that can be provided by long-term IoT-based maternal monitoring systems. Section 4 discusses the presented architecture and our implementation for the high-risk pregnancy study. Evaluation of the presented system and discussion of the technical challenges are provided in Section 6. Finally, Section 7 concludes the paper.

## 2. Related Work

In this section, we briefly discuss state-of-the-art IoT-based healthcare systems designed for maternal health monitoring.

There is a large body of literature introducing remote monitoring services in pregnancy. Most of these studies use questionnaires to track mother’s health condition [32] or investigate certain issues or health problems during pregnancy such as hypertension [23,33], preterm birth [14], gestational diabetes [34] and sleep disturbances [21]. Few works have exploited long-term IoT-based health monitoring in pregnancy and postpartum.

Some studies tailored self-reports along with wearable devices for a short period of time to monitor pregnant women. For example, Tsai et al. [15] investigated the correlation of sleep problems and health-related quality of life using questionnaires and wrist actigraphy (a non-invasive method for monitoring sleep and activity) for seven days in each trimester. Other studies track different parameters in pregnant women, such as blood pressure, weight and blood glucose, to monitor hypertension [19] and diabetes [34]. In [19], mothers with gestational hypertension were recruited to use home blood pressure monitoring. If values were higher than a specified threshold, mothers would be referred to visit hospitals.

Smartphone applications have been introduced to notify mothers in case of high-risk situations. Krapf et al. [35] developed a mobile application to collect recorded values of blood pressure and weight from mothers and notify mothers in case of abnormality detection. Allahem et al. [14] proposed a framework to monitor pregnant women with high risks of premature birth. They aimed to reduce preterm birth by collecting uterine contractions through a body sensor and informing women via a mobile application if the collected information was above some personalized thresholds. In [23], the authors used a smartphone-based system enabled by a Naive Bayes Classifier, performing real-time decision-making.

Wearable devices have also been utilized to collect maternal health parameters continuously. In [33], a model is proposed for hypertension monitoring during pregnancy. In this study, a commercial wristband was leveraged to monitor heart rate, step count and sleep. The proposed model was evaluated in a healthcare center for three months. Pregnant women were satisfied with this model, as they could monitor their own health in a non-invasive way. In [21], the authors presented an IoT-based monitoring system for objective sleep quality assessment. They used a smart wristband to collect sleep information from mothers continuously and provide a personalized model indicating the degradation of sleep quality according to each person’s data. Kumar et al. [13] proposed an architecture for health monitoring during pregnancy, considering the needs for adaptation of the system based on collected health data.

These studies utilize IoT-based systems for maternal health monitoring. However, they are restricted to *specific health issues* in *a short period of time* or performed with *limited data collection* methods. An IoT-based maternal health monitoring system is required, enabling continuous and long-term monitoring of a mother’s health conditions. Such monitoring should provide a holistic view of mothers’ health, promoting healthy lifestyles in pregnancy and reducing the risk factors for health in pregnancy. For example, preterm birth complications are the most frequent cause of neonatal death [36], occurring in most cases without pre-known cause [37]. Such health issues could be tracked and uncovered by collecting and analyzing various parameters such as stress-related factors and behaviors in pregnancy [36].

Moreover, there are studies to investigate the feasibility of long-term maternal monitoring systems. Marko et al. [20] assessed the use of a mobile application for six months during pregnancy. The mobile application was connected with a blood pressure device and a digital weight scale. This monitoring was conducted for eight women with low-risk pregnancies. Grym et al. [6] also evaluated the feasibility of using a smart wristband by conducting a case study on maternal health to monitor 20 pregnant women for seven months.

There is a need to investigate the use of maternal health monitoring systems from different perspectives [6,28,38,39]. These systems should provide a high level of quality attributes (e.g., feasibility, reliability and energy efficiency) to satisfy the requirements of the long-term monitoring and improve user experience. In this regard, the technical and practical challenges should be evaluated for health monitoring scenarios outside clinical settings. Moreover, AI and machine learning-based methods should be utilized in a holistic way to assess the quality of data, impute missing data and make personalized maternal health monitoring.

## 3. Long-Term IoT-Based Maternal Monitoring Services

This section outlines long-term maternity care services, i.e., physical activity monitoring, sleep monitoring and stress monitoring, which could be offered by IoT-based systems for maternal remote health monitoring.

### 3.1. Physical Activity Monitoring

Moderate physical activity is vital for a pregnant woman’s general well-being and quality of life [40]. In addition, physical activity reduces the risks of obstetric complications [41]. It is well known that the level of physical activity decreases as pregnancy progresses [42] and subjective reports tend to overestimate the volume and intensity of activity [43]. Continuous objective monitoring would provide individualized and detailed information about the woman’s physical activity, and her counseling could be tailored according to her needs. Therefore, monitoring would support both the care provider and the pregnant woman.

### 3.2. Sleep Monitoring

Sleep disorders are pervasive in pregnant women, and the most prevalent period is during the third trimester [44]. Frequent urination and difficulty in finding a comfortable position disturb sleep [45]. Sleep disorders during pregnancy have been associated with the risk of preterm birth [46], gestational hyperglycemia [47] and mood disorders [48]. Total sleep time decreases gradually during pregnancy, but some women experience acute sleep deprivation in labor and the early postpartum period [48]. Similar to monitoring of physical activity, continuous sleep monitoring would provide individualized sleep information, and tailored sleep counseling would support a healthy circadian rhythm, which, by implication, could also support diet and weight management [49].

### 3.3. Stress Monitoring

Pregnant women can experience stress about daily hassles but also related to the pregnancy itself: e.g., due to physical symptoms, bodily changes or the health of the fetus. A high level of maternal prenatal stress seems to increase the risk of adverse pregnancy outcomes such as hypertensive disorders, depression and preterm birth [50,51]. Based on recent studies, it is also known that stress during pregnancy may have long-term impacts on the infant and mother–infant interaction [52,53]. Thus, these issues should not be underestimated in maternity care. Continuous long-term monitoring of stress might help pregnant women to identify and possibly even manage their stress better. Maternity care providers could also target the right interventions for women whose level of stress is high.

## 4. Maternal Health and Well-Being Monitoring System

**Figure 1:**
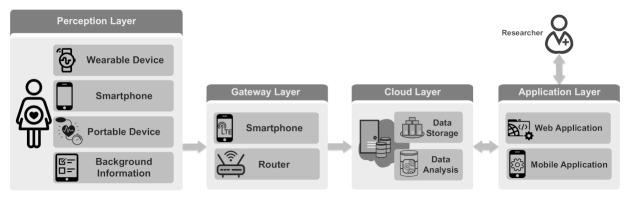
IoT-based maternal health monitoring system.

In this section, we present an IoT-based system designed for *long-term* maternal health monitoring during pregnancy and postpartum. The presented architecture is shown in Figure 1. This system enables the monitoring of pregnant women *continuously* and integrates *subjective* and *objective* data collected from the mothers, thus providing a holistic view of mothers’ conditions. The monitoring system consists of four layers: perception layer, gateway (i.e., fog/edge layer) layer [54,55], cloud layer and application layer. In the perception layer, physiological well-being information is collected from mothers, exploiting various types of sensors. Collected data are sent to the cloud layer through the gateway layer. The cloud layer stores and analyzes the data and provides processed data for visualization. The application layer visualizes health information to the users. It also enables the researchers to communicate with the mothers. In the following, we briefly describe the four layers.

### 4.1. Perception Layer

The perception layer collects the mother’s health data using several types of data sources. The data sources can be divided into wearable devices, smartphones, periodic portable devices and background information.

#### 4.1.1. Wearable Devices

Wearables such as smart rings, smartwatches and Holter monitors are the main data collectors in such IoT systems for objective data acquisition. These devices contain sensors to measure bio-signals continuously, e.g., Photoplethysmogram (PPG) (PPG is a non-invasive optical method which can be used for acquiring heart rate and heart rate variability) [56], accelerometer and gyroscope. The signals are then analyzed to obtain various health-related parameters—such as stress, sleep and physical activity—providing maternity care services. In maternal IoT-based systems, the objective is to monitor health parameters for a long period of time, so the selected wearable device should be small, easy-to-use and energy-efficient while satisfying different quality attributes such as feasibility and usability. Moreover, the device should be feasible to use even if the participant gains weight rapidly or has swelled.

#### 4.1.2. Smartphone

Smartphones can perform data collection in the monitoring, as they include various sensors such as accelerometer, gyroscope and magnetometer. These sensors are used to track physical activity and sleep. Moreover, patterns of smartphone usage, e.g., call duration, number of text messages, etc., can be another data source showing some psychological problems [57].

In addition, smartphones can act as a bridge between the users and health providers, enabling two-way communications. In this regard, smartphones are tailored to perform subjective data collection, using Internet-based self-report questionnaires and momentary ecological assessments. Such data can be used for screening and diagnosis purposes. To enable self-reported data collection, a mobile application is needed, by which questions are delivered to the users at different time intervals (e.g., daily or weekly). Moreover, users could send other health information, ask technical questions about the system or communicate with a nurse/physician when needed.

#### 4.1.3. Portable Devices for Periodic Monitoring

Periodic/episodic portable devices refer to devices used to measure physiological parameters once a day or once a week. Some physiological parameters, such as weight, change slowly. In addition, parameters such as blood pressure and blood glucose cannot be measured continuously in a noninvasive and clinically reliable way [58,59]. Periodic portable devices measure such parameters and transmit the values directly to the cloud, or users can manually report the values via the mobile application.

#### 4.1.4. Background and Demographic Information

Background and demographic information is another data source containing mothers’ information, which could assess the risk of health complications during pregnancy and postpartum. For example, having a previous miscarriage or preterm birth increases the preterm birth risk in future pregnancies [60]. In addition, ethnicity, age, weight before pregnancy, diagnosed diseases and mother’s lifestyle can increase the risk of pregnancy complications, thus needed for personalization purposes. This information can be collected from mothers through the mobile application or hospital information services if proper agreements and consents are provided.

### 4.2. Gateway Layer

The gateway layer provides interoperability between the perception and the cloud layers. The gateway’s primary purpose is transmitting collected data from mothers’ personal area network to the cloud server. The gateway can be a router or a smartphone. Moreover, smart gateways [61] can be used to provide other services at the edge, such as local data analysis, data compression, embedded data mining, reliability and security.

### 4.3. Cloud Layer

The cloud layer consists of a remote server that receives collected data from the perception layer through the gateway layer. It provides secure central storage to store a massive volume of health data. In addition, it enables performing big data analytic and machine learning algorithms to find trends and anomalies in the collected data. It provides personalized health monitoring and alarm notifications by analyzing individuals data. Moreover, the cloud server can be used as a server part in a client-server paradigm to visualize the data for the caregivers and mothers.

### 4.4. Application Layer

The application layer provides the interface to the end-users for easily interacting with the system. This layer provides web applications and cross-platform mobile applications for monitoring and visualizing data, as well as means for two-way communication between the researchers and mothers. The web application provides a dashboard for caregivers to monitor and interact with mothers’ in real-time. In addition, mothers can use the cross-platform mobile application to monitor their own health data, which can lead to better self-management.

## 5. Implementation

In this section, we describe the setup and our case study of a IoT-based maternal health monitoring system.

### 5.1. Perception Layer

Data sources used in our implemented system includes a smartwatch, a cross-platform mobile application, a blood pressure device, and background information.

#### 5.1.1. Wearable Devices

The Samsung Gear Sport smartwatch [62] is selected in this study, considering access to raw data, battery life, configurability of the data collection, adequate built-in memory and waterproofness. The watch includes one built-in inertial measurement unit (IMU) and one PPG sensor.

The Gear Sport watch runs an open-source Tizen operating system (OS) [63], which enables us to develop our smartwatch applications for a customized data collection. We developed several data collection services in the C programming language, which run with no user interaction. These services can be used for any wearable or smartwatch that runs Tizen OS, such as the Samsung Galaxy, Active and Active2 smartwatches [64].

In this monitoring, the PPG signal is acquired to extract heart rate and heart rate variability (HRV) parameters. We programmed the watch to collect the PPG signal for 12 min every second hour, considering the watch’s battery life (see Section 6.3). Acceleration data and daily activity data (e.g., step counts) provided by the watch are also acquired to track participants’ physical activity and sleep.

Moreover, we develop an application enabling users to upload collected data to the server through the WiFi connection. A data compression method is also used to reduce bandwidth usage. During the monitoring, the participants are asked to continuously wear the device and upload the collected data to the server using the application frequently (e.g., daily).

#### 5.1.2. Smartphone

**Figure 2:**
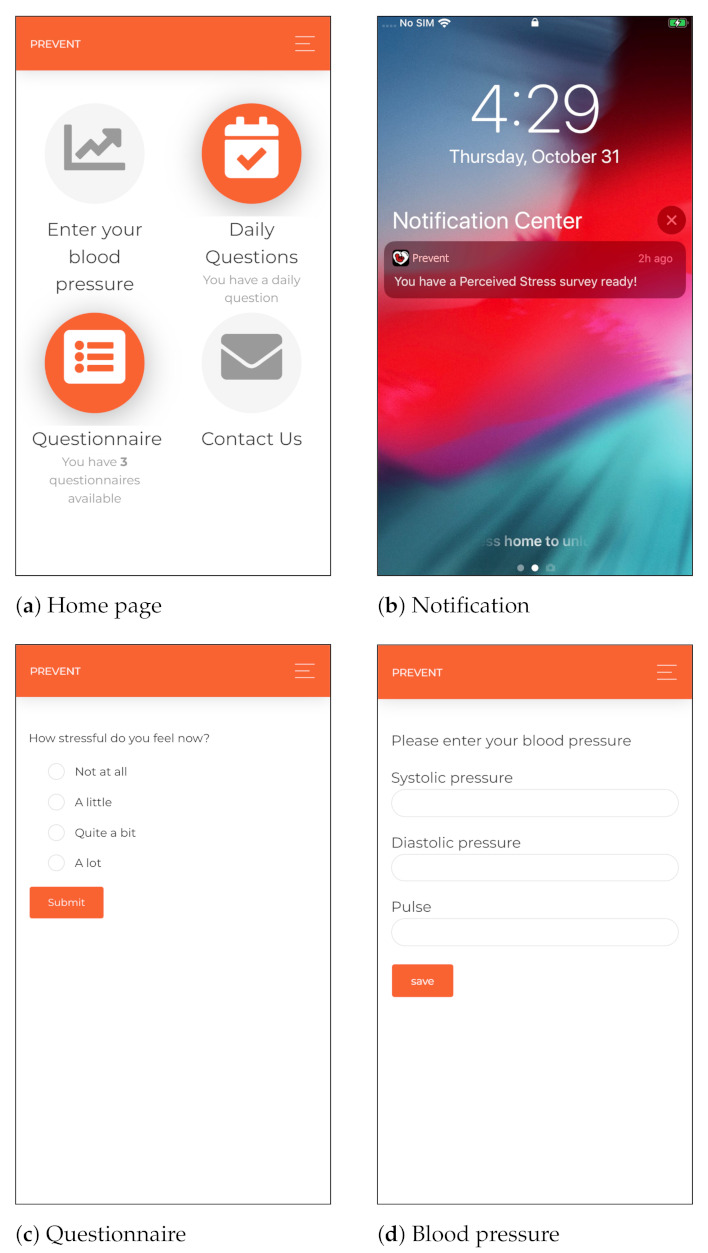
Different interfaces of the cross-platform mobile application leveraged in our monitoring.

We developed a cross-platform mobile application for smartphones to collect self-report data, including momentary ecological assessments using random, daily and weekly questionnaires. The mobile application includes components for delivering health parameters (such as blood pressure), requesting technical supports, collecting background information, sending push notifications and reminders and providing communication services if the user has any concerns regarding her health condition. The cross-platform mobile application is developed using the Angular 2 technology [65] and Cordova [66], which are open-source and platform-independent frameworks. Moreover, considering the security aspects of the monitoring, the participants are authenticated using token-based authentication and authorized to access the mobile application. Different interfaces of the proposed cross-platform mobile application are shown in Figure 2.

#### 5.1.3. Portable Devices for Periodic Monitoring

The participants were asked to measure their blood pressure at least once a week and send the data through the mobile application to the server, as shown in Figure 2d. In this regard, an OMRON M3 Intellisense blood pressure device [67] was given to each participant at the beginning of the monitoring. The selected blood pressure device is clinically validated. Moreover, the cuff supports the 360 accuracy feature, providing accurate reading regardless of cuff placement on the arm. This feature enables non-expert users to collect accurate measurements [68].

#### 5.1.4. Background and Demographic Information

Using the cross-platform application, we sent a self-report questionnaire to the participants to collect their background information. The questionnaire is designed to gain insights into their diagnosed diseases, previous miscarriage or preterm birth, lifestyle and perceived stress.

### 5.2. Gateway Layer

There are two types of gateway devices used in this monitoring. The first device is the smartphone. Our mobile application is a client–server application that uses the smartphone’s Internet connectivity to send data from the application to the server. The second gateway device is a WiFi router, providing Internet connection for the smartwatches during the monitoring.

### 5.3. Cloud Layer

We used Apache 2 [69], an open-source, cross-platform web server, and Flask [70] for developing our server. Flask is an open-source Python WSGI (Web Server Gateway Interface) framework that provides scalability and flexibility. It also speeds up the development. We exploited the MongoDB [71] to store the data. MongoDB is a NoSQL database that provides flexibility in the variety and types of stored data. An SSL API (Secure Sockets Layer Application Programming Interface) was also utilized to provide secure communication.

Our server provides user management, data management and data analysis. The user management module is responsible for creating new user accounts, modifying current users, assigning proper access levels to the users and allocating a set of questions to the users. In this setup, an authorized user can add, modify and delete the questions and schedule a time for certain notifications and reminders. The data are stored anonymously in the server to ensure the user’s privacy.

The data management module receives data from the mothers through the mobile application and the wristbands. The server implements an authentication mechanism. Then, the validity of the received data is checked. The user is notified to re-upload the data in case of errors occurring. No personal data are sent to the server concerning the users’ privacy. Moreover, the users need to be authenticated and authorized for accessing the data.

The data analysis module is responsible for analyzing the collected subjective and objective data in this monitoring system. This module provides stress, physical activity and sleep monitoring services.

Stress monitoring service in this system is provided by monitoring heart rate and HRV parameters. Studies have shown that the HRV parameters are linked to the autonomic nervous system activity changes associated with the level of stress [72]. Mental stress increases the LF (power in low-frequency range) and decreases the HF (Power in high-frequency range). Psychological stress is also significantly associated with an increase in the LF/HF ratio. Another important HRV parameter is SDNN (standard deviation of all normal IBIs), which is an index of resilience against stress. Moreover, in stressful conditions, RMSSD (root mean square of the successive differences), AVNN (average of normal IBIs) and LF/HF decrease and HF value increases in the short-term HRV measurements (see more details in [72,73]). Different HRV parameters used by this IoT-based system for stress monitoring are presented in Table 1. Therefore, the PPG signal is utilized to derive heart rate and HRV parameters. The heart rate is extracted by counting the number of heartbeat peaks in the signal. Moreover, we obtain the HRV parameters by extracting the variation of inter-beat interval (IBI) in the PPG signal. The IBI is the duration of two successive heartbeat peaks in the signal.

**Figure 3:**
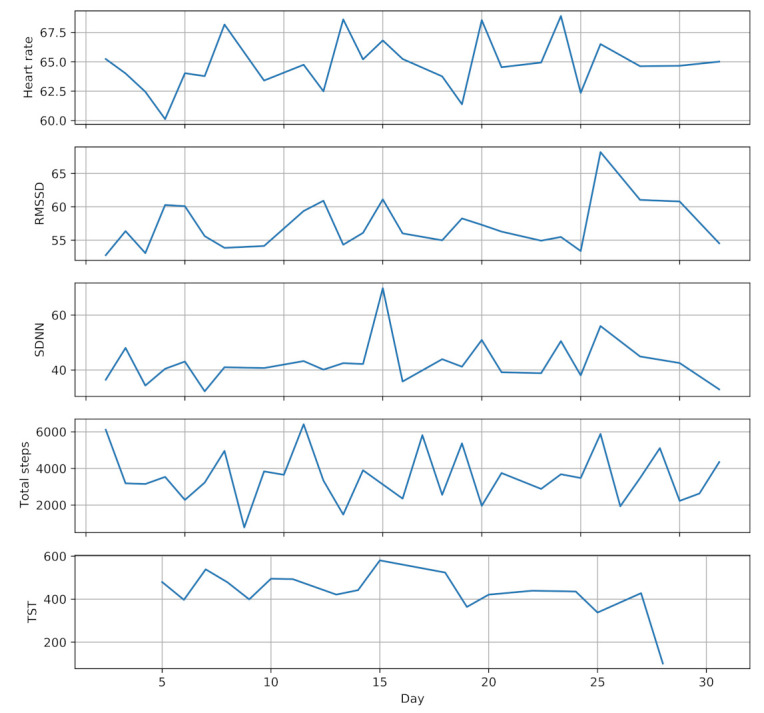
A one month sample of collected data from one participant during pregnancy.

Sleep monitoring service uses hand movement and step counts data provided by the smartwatch to extract sleep parameters, including total sleep time (TST), sleep efficiency (SE) and wake after sleep onset (WASO) [74]. Physical activity monitoring service also leverages step counts and wearing time data to estimate the daily physical activity and sedentary time of the participants. Figure 3 shows a one month sample of the collected data from one participant (randomly selected) during pregnancy.

**Figure 4:**
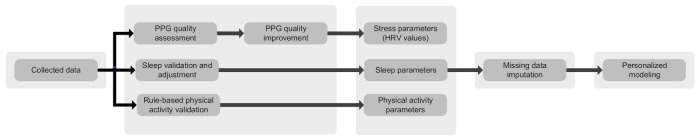
Data analysis pipeline.

The data analysis module consists of various artificial intelligence and machine learning algorithms to assess the quality of data, detect anomalies, find trends and create personalized models, to mention a few [21,30,31]. The data collection is performed in everyday settings while the mothers engage in various physical activities. Therefore, the collected data (particularly PPG) are susceptible to environmental noises and motion artifacts. Quality assessment methods are utilized to differentiate reliable and unreliable data [30]. Consequently, reliable data are used for further analysis and decision-making. Moreover, machine learning algorithms are exploited to train patients’ models, by which trends and changes are evaluated throughout the pregnancy and postpartum [21]. We integrate previously proposed methods by the authors in a holistic way to provide a pipeline for data processing, deep learning-based PPG quality assessment [30], machine learning-based missing data imputation [31], personalized modeling, and anomaly detection [21]. Figure 4 depicts the data analysis pipeline in more detail.

### 5.4. Application Layer

**Figure 5:**
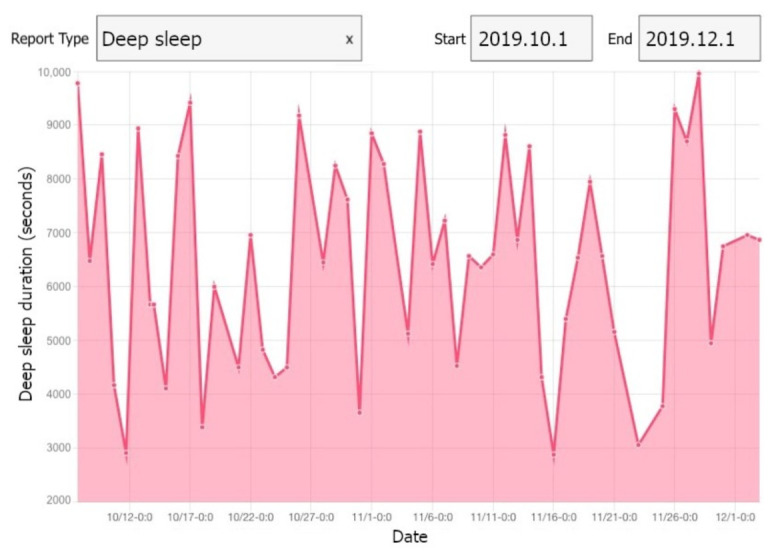
A view of our web application showing deep sleep of one participant.

We developed a web application using Angular 2, by which we were able to reuse the components of our mobile application in the development. The implemented web application enables the researchers to monitor the collected data. The trends and changes can be observed via different daily/weekly plots in the web application. A view of our web application is illustrated in Figure 5.

## 6. Evaluation and Discussion

In this study, 28 women with high-risk pregnancies were monitored during pregnancy and three months postpartum using the presented system. We evaluate the presented system in terms of feasibility, reliability of the measurements and energy consumption. Moreover, we discuss the potential, limitations and practical challenges of our system. Finally, we investigate the possibility of integrating the presented system into the current healthcare system.

Study Design and Participants: This study was conducted on women with high-risk pregnancies, exploiting the presented IoT-based system. The participants were recruited via advertisements in maternity clinics in Southwestern Finland and social media in 2019. Interested pregnant women contacted the researchers via email. The eligibility criteria for participants were: (1) greater or equal to 18 years of age; (2) 12–15 gestational weeks; (3) singleton pregnancy; (4) previous late miscarriage (12–22 gestational weeks) OR previous preterm birth (22–36 gestational weeks); and (5) ability to understand Finnish.

In addition, the participants had to have a smartphone (Android or iOS) and accept wearing a smartwatch from the recruitment until three months after the delivery. The eligible women were asked to participate in a face-to-face meeting with researchers, in which the details of the study procedures were provided. After the written informed consent, the devices, including a smartwatch and a blood pressure device, with instructions were delivered to the participants. In addition, a mobile application developed for this study was installed on their smartphones. Thirty-two pregnant women with high-risk pregnancies were recruited in this study. Four women withdrew from the study during the data collection period. Thus, the final sample size in this study was 28 pregnant women. Participants had a median of 13.4 weeks of gestation at the beginning of the monitoring.

### 6.1. Feasibility

The feasibility of using the IoT-based system for a long period of time (i.e., nine months) and usability of the data collectors for mothers are important considerations in such long-term studies. In the following, we investigate the feasibility of wearing the smartwatches and using our mobile application to answer the daily questions and send the blood pressure measurements, considering the average usage of devices.

#### 6.1.1. Wearable Device Usage

In this section, we examine the average wearing time of the smartwatch per day during pregnancy and postpartum, showing its usability in our study.

**Figure 6:**
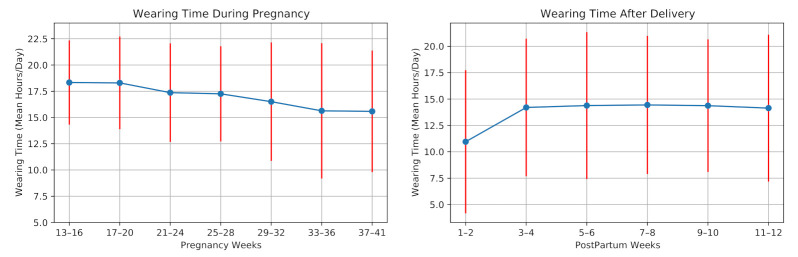
Smartwatch wearing time during pregnancy and postpartum of the 28 high-risk pregnant women.

The average daily wearing-time during pregnancy and postpartum for all participants is shown in Figure 6. Six participants experienced preterm birth as their pregnancies lasted fewer than 37 weeks. One participant was not allowed to use the wristband at work, thus having a minimum average wearing time. Three participants were hospitalized due to pregnancy complications for several days, having a restriction in using the smartwatch. Moreover, the data collection was interrupted due to technical issues, e.g., server failures. The data of twelve participants were lost for (on average) four days.

The watch wearing-time decreased on average during the pregnancy. In the postpartum period, the wearing-time increased for most of the participants during the first weeks. Two participants could not use the device after the delivery due to the restrictions of the Neonatal intensive care unit (NICU). The average wearing time during pregnancy was 17.01 ± 4.20 h/day, and it decreased to 13.72 ± 5.71 h/day after the delivery.

In a feasibility study of using smart wristband during pregnancy, Grym et al. [6] showed that the average wearing time during pregnancy was 17.3 h/day and for one month after delivery was 14.4 h/day. Our results are in accordance with this study. However, the wearing time in our study is slightly lower than their findings. This can be explained due to the hospitalization of the mothers with high-risk pregnancies in our study. Consequently, the data collection using the smartwatch is feasible during pregnancy and postpartum.

#### 6.1.2. Cross-Platform Mobile Application Usage

**Figure 7:**
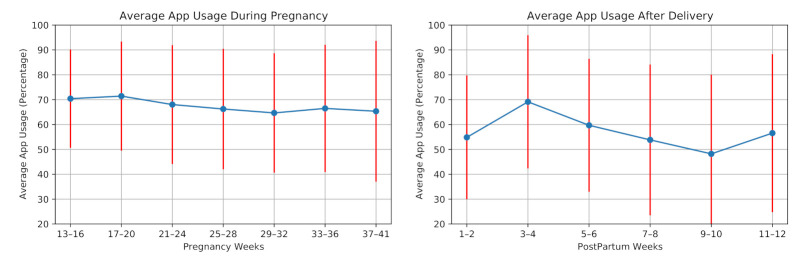
Average mobile application usage (participants engagement in answering daily questions) during pregnancy and postpartum of the 28 high-risk pregnant women.

We investigate the average usage of our cross-platform mobile application in the monitoring from two aspects: answering the daily questionnaires and uploading blood pressure values through the mobile application. Figure 7 shows the average application usage for the participants during pregnancy and postpartum. On average, the application usage decreased during pregnancy. Similar to the wearing time of the smartwatch, application usage increased after the second week of the postpartum period. However, it decreased in the following weeks. In this study, the participants answered 5493 daily questions, including 3879 answers during pregnancy and 1614 answers in postpartum. The average application usage for answering daily questions was 67.5% of the days in pregnancy and 57.0% of the days in postpartum.

**Figure 8:**
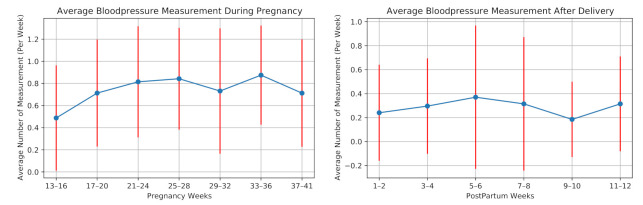
Weekly average number of measuring blood pressure of the 28 high-risk pregnant women during pregnancy and postpartum.

The participants were also asked to measure their blood pressure once a week. Figure 8 illustrates the average number of blood pressure measurements per week for all the participants during our study. One participant measured her blood pressure almost every day, as she had preeclampsia (a disorder of pregnancy associated with high blood pressure) in her previous pregnancy. We remove this participant before averaging the number of measurements considering this high measurement rate as an outlier.

On average, the participants (excluding one with previous preeclampsia) measured their blood pressure 0.74 times in a week during pregnancy and 0.29 times per week after delivery. Ten participants did not perform any blood pressure measurement after delivery.

To the best of our knowledge, the feasibility of daily self-report questionnaires using a mobile application during pregnancy and postpartum has not been investigated. Our results show 5.44 times/week of blood pressure measurement and mobile application usage, which is similar to results in [20]. In consequence, the data collection using the mobile application in daily usage is feasible during pregnancy and postpartum.

### 6.2. Robustness and Reliability of Measurement

Remote monitoring systems need robust and reliable measurements (i.e., data collection) to provide accurate and reliable decision/risk estimation. The PPG signals collected from wristbands are highly affected by motion artifacts and environmental situations. These noises can result in low-quality signals, which may lead to unreliable health risk decisions. Unfortunately, such low-quality PPG signals are highly probable in these long-term health monitoring systems, as the users are engaging in various physical activities. Therefore, it is essential to ensure an acceptable quality of PPG signals in such systems.

In our setup, the Gear sport smartwatch provides raw PPG signals with a maximum sampling frequency of 20 Hz. The signals are processed to obtain the heart rate and HRV parameters of the mothers. We customized our watch to perform reliable measurements by configuring the frequency and duration of the data collection. It should be noted that, in such long-term monitoring, there is a trade-off between the availability/reliability of the measurements and the energy consumption of the data collectors.

#### 6.2.1. Duration of PPG Signal Recording

As mentioned, the PPG signal is collected to obtain the heart rate and HRV parameters of the participants. The duration of each signal record highly impacts the accuracy and reliability of the heart rate and HRV parameters. An accurate heart rate is obtained using a 60-s window of PPG signal. However, according to the literature, there are different standards of measurement for the HRV parameters [75,76,77]:

- Twenty-four-hour recordings (referred as long-term HRV analysis) are used to derive HRV parameters.
- Five-minute recordings (referred as short-term HRV analysis) are utilized to obtain HRV parameters.
- Less-than-five-minute recordings (referred as ultra-short-term HRV analysis) are exploited to extract some of the HRV parameters.

In an ideal situation, the 24-h recordings should be performed in the maternal monitoring, reflecting the overall changes of the heart rate under non-specific conditions. Unfortunately, the 24-h PPG signal collection is inapplicable in existing wearable devices due to battery constraints. The PPG signal is acquired by emitting light (i.e., green light in our device) to the skin surface and collecting the light reflection. Sensing energy consumption, including the photoemitter (LED) and photodetector, considerably affects the watch’s battery life (see more details in Section 6.3).

Therefore, we programmed the watch to collect PPG signal in a regular and consistent manner, enabling the 5-min recordings throughout the monitoring. The watch was configured to record the PPG signal for 12 min every second hour, including 2 min of sensor calibration (i.e., unreliable data) and two 5-min recordings. In this setup, we selected two consecutive 5-min recordings to remove low-quality PPG signals in each record, reducing the impact of noise and motion artifacts on the HRV parameters to ensure the reliability of the collected HRV parameters by collecting sufficient PPG signals for analyses.

#### 6.2.2. Sampling Frequency of the PPG Signal

The sampling frequency of the PPG signal also influences the accuracy and reliability of the heart rate and HRV values. Heart rate can be obtained by extracting cardiac frequency (i.e., 0.5–3 Hz) from the PPG signal exploiting filter-based techniques. However, a higher sampling frequency is required to derive HRV parameters. The accuracy of these parameters was investigated in the literature by comparing different PPG signals (5–10,000 Hz sampling frequency) with an Electrocardiogram (ECG) signal (10,000 Hz sampling frequency) as the golden standard [78]. It was shown that HRV parameters—related to the stress level such as RMSSD, LF, HF and AVNN—necessitate PPG signal collection with at least 20 Hz as the sampling frequency.

In our setup, we selected the sampling frequency of the PPG signals as 20 Hz to guarantee an acceptable accuracy of heart rate and HRV parameters. Subsequently, we can reliably obtain stress-related HRV parameters using this system.

### 6.3. Energy Consumption

Energy consumption is an important issue in a remote health monitoring system consisting of devices with limited batteries. In such devices, battery-powered sensors collect and transmit the data continuously, consuming considerable energy. Particularly, in long-term monitoring, the battery life of wearables (i.e., the time a device works before its battery requires to be recharged) could significantly impact the system’s feasibility and usability. There is a wide variety of energy efficiency methods in the literature for addressing energy consumption in IoT-based systems [79,80,81,82].

**Figure 9:**
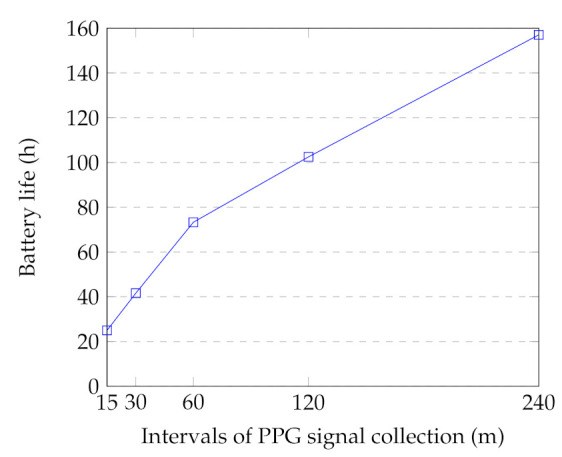
Battery life time of the watch for different intervals. Each interval contains 12 min of PPG signal collection.

One of the drawbacks of the PPG method (including a light source and a light sensor) is its high energy consumption [26,83]. In our setup, the battery life of the smartwatch is highly affected by: (1) the PPG collection duration (discussed in Section 6.2.1); (2) the PPG sampling frequency; and (3) the time intervals between the PPG collection. We examined the battery life of the smartwatch by collecting 12 min of PPG signals in different intervals. In this regard, three smartwatches were utilized to record the signals. The watches were in flight mode with no movement during the tests to reduce the usage bias. Figure 9 indicates the average results for different intervals. In the case of PPG signal collection in the 15-min interval, the battery lasted only 25 h. However, the battery life increased to 157 h in the 240-min intervals. In our case study, we selected 2-h intervals to guarantee 2–3-days of battery life of the watch during the monitoring. It should be noted that the device usage also reduces the battery life due to other watch’s functions, including Bluetooth connection with the smartphone and the physical activity application. This battery life results in feasible long-term monitoring and acceptable effort in everyday settings data collection.

### 6.4. Practical Challenges

Various practical and technical challenges arise in the era of long-term remote health monitoring. These challenges can be investigated from different perspectives, such as user experience and integration of the system with newer technologies.

One major challenge is keeping the users motivated in the long-term. Several studies show that social interaction, personalized monitoring, usability in daily life settings, and the design of the long-term intervention enhance user experience and keep the participants motivated to continue the monitoring [24,84,85].

For usability, the wearable devices in such systems should require reasonable effort to use and be comfortable in everyday life settings [24]. Moreover, the system should have satisfactory monitoring functions and interfaces. For this purpose, we chose a wearable device that can be easily used in indoor and outdoor activities. The monitoring services offered by the presented system are known to be effective in improving pregnant women’s well-being (see Section 3). Our monitoring applications also require minimum interaction with the participants. However, the participants need to upload the data manually. We provide technical supports to the participant in the case of technical problems. Moreover, we notified the participants if they had forgotten to upload the data for two weeks. In future work, data uploading will be automatic and real-time to improve user experience.

Another effort required by the user is charging the device. We discuss this in Section 6.3, optimizing user effort and data collection. Frequent charging of the device also increases the missing data and affects the reliability of the system.

Moreover, in the long-term, the technology evolves, and the system needs to be integrated with newer technologies. We designed and developed our software programs in the server and the application (client) sides using the best practice software development methodologies and frameworks, such as RESTful API, Flask, Angular 2 and MongoDB. This approach enables us to integrate our system with newer technologies and devices easily. Moreover, the wearable device used in this system runs Tizen OS, which supports backward compatibility. Therefore, our developed applications for the smartwatch could work adequately regardless of minor and major updates of the OS. We also developed an application for restarting all of our monitoring applications on the smartwatch. The participants could use the app whenever they have some troubles using the watch or uploading the data. In future work, we will integrate Validic API in our system to easily collect data from various wearable and monitoring devices [86].

### 6.5. Integration to The Current Healthcare System

Current maternity care in Finland is based on appointed meetings and physical measurements by the healthcare professionals. The remote monitoring system described in this paper could bring a new element to traditional maternity care. Monitoring would enable pregnant women to control the measurements and, by implication, possibly engage the women better in their self-care [87,88]. Moreover, this system would also change the work of maternity care professionals; thus, tight collaboration with them will be needed if establishing new elements into their work. Based on the previous study, maternity care professionals, as well as pregnant women, are interested in using remote monitoring, at least with certain groups of women. The safety of the pregnant woman and her unborn infant was considered the most serious challenge in remote monitoring.

Importantly, the new system should be assimilated as part of the previous system to enhance the implementation [39]. Globally, the WHO recommends using digital technologies to enhance the coverage and quality of health services [89]. In developing countries, digital technology also plays a significant role in supporting services for maternal and child health. Thus, the IoT systems could enable countries of all kinds to develop their care [90].

Our implemented IoT-based system was limited to the health monitoring of pregnant women with no feedback to the mothers. For future work, we plan to perform risk prediction and detect various health problems during pregnancy and postpartum. Moreover, we need to evaluate this system for personalized intervention to reduce the risk or prevent adverse health problems in pregnancy. We also considered the interoperability of our collected data with the clinical healthcare system. Our applications used RESTful API, presenting data in JSON format. JSON files can be easily converted to other types of data. In future work, we will consider providing data in HL7 format [91] for integration with the clinical system.

## 7. Conclusions

Maternal health monitoring is important to ensure the health and well-being of the mother and her child, as many health complications occur during pregnancy with a lifetime effect on their health. In the literature, some studies exploited IoT-based systems for maternal monitoring, although they were limited to specific health problems, short-term data collection or self-report questionnaires. In this study, we first presented an IoT-based maternal monitoring system, providing services such as physical activity, sleep and stress monitoring throughout pregnancy and postpartum. Then, we implemented and evaluated the presented system with a real human subject study on high-risk pregnant women. This system utilized various data collectors, including a cross-platform mobile application and a smartwatch, to collect bio-signals and self-report data. The collected data were stored and analyzed in the cloud server. We discussed the feasibility of the system, considering the usage of the smartwatch and the mobile application. Our results show that participants, on average, used the smartwatch 17.01 ± 4.20 h/day during pregnancy and 13.72 ± 5.71 h/day in postpartum. The average application usage for answering daily questions was 67.5% of the days in pregnancy and 57.0% of the days in postpartum. These results show the feasibility of the implemented system in terms of interacting with the system (mobile application usage and smartwatch wearing time by participants). We also evaluated the system in terms of the energy efficiency of the smartwatch and the reliability of the collected data. Our findings show acceptable energy consumption of the watch in long-term monitoring as well as a reliable PPG-based analysis. Moreover, we investigated the integration of the presented system with the current healthcare system. As future work, we will address the energy efficiency and reliability by proposing an adaptive data collection technique leveraging the participant’s activity, health status and stress level. Moreover, we will consider adding other services to the monitoring system such as diet and preeclampsia monitoring and providing feedback to the users. We will also provide APIs enabling adding several wearable devices and interoperability with clinical healthcare systems. In addition, future work should consider evaluating attributes (e.g., latency and availability) related to the real-time services or intervention.

## Figures and Tables

**Table 1:** HRV parameters.

Variable	Units	Description
NN interval	ms	Normal inter-beat interval
RMSSD	ms	The square root of the mean of the sum of the squares of differences between adjacent NN intervals
AVNN	ms	Average of NN intervals
SDNN	ms	Standard deviation of all NN intervals
LF	ms${}^{2}$	Power in low-frequency range (0.04–0.15 Hz)
HF	ms${}^{2}$	Power in the high-frequency range (0.15–0.4 Hz)
LF/HF	-	LF/HF

## Data Availability

Data sharing not applicable.
